# Public Health Approaches to Type 2 Diabetes Prevention: the US National Diabetes Prevention Program and Beyond

**DOI:** 10.1007/s11892-019-1200-z

**Published:** 2019-08-05

**Authors:** Stephanie M. Gruss, Kunthea Nhim, Edward Gregg, Miriam Bell, Elizabeth Luman, Ann Albright

**Affiliations:** 1 Division of Diabetes Translation, Centers for Disease Control and Prevention, 4770 Buford Hwy., Mailstop F75, Atlanta, GA 30341 USA; 2 Department of Epidemiology and Biostatistics, School of Public Health, Imperial College London, London, UK

**Keywords:** Diabetes prevention, National Diabetes Prevention Program, Type 2 diabetes, Diabetes policy, Obesity, Diabetes prevention program

## Abstract

**Purpose of Review:**

This article highlights foundational evidence, translation studies, and current research behind type 2 diabetes prevention efforts worldwide, with focus on high-risk populations, and whole-population approaches as catalysts to global prevention.

**Recent Findings:**

Continued focus on the goals of foundational lifestyle change program trials and their global translations, and the targeting of those at highest risk through both in-person and virtual modes of program delivery, is critical. Whole-population approaches (e.g., socioeconomic policies, healthy food promotion, environmental/systems changes) and awareness raising are essential complements to efforts aimed at high-risk populations.

**Summary:**

Successful type 2 diabetes prevention strategies are being realized in the USA through the National Diabetes Prevention Program and elsewhere in the world. A multi-tiered approach involving appropriate risk targeting and whole-population efforts is essential to curb the global diabetes epidemic.

**Electronic supplementary material:**

The online version of this article (10.1007/s11892-019-1200-z) contains supplementary material, which is available to authorized users.

## Introduction

Worldwide, it is estimated that 425 million adults (20–79 years) have diabetes, projected to reach 629 million by 2045 [1•]. In the USA, about 30 million adults (18 years and older) have diabetes, or 12.2% of the adult population [2]. Diabetes was estimated to cost $727 billion in 2017 in health expenditures worldwide [1] and $327 billion in 2017 in total economic costs in the USA [3•]. Increases in diabetes prevalence have led to increases in related complications, such as cardiovascular disease, visual impairment and vision loss, lower extremity amputations, end-stage renal disease, disability, and premature mortality [2].

The majority of diabetes is type 2, which generally follows a period of prediabetes, a condition where blood glucose levels are higher than normal, but not high enough for a type 2 diabetes diagnosis [2]. In the USA, risk factors for prediabetes and type 2 diabetes include being overweight or having obesity; having a racial/ethnic background that is African-American, Hispanic/Latino, American Indian, Asian American, or Pacific Islander; having a parent or sibling with diabetes; having hypertension; having a history of gestational diabetes; and living a sedentary lifestyle. An estimated 353 million adults worldwide [1]—84.1 million in the USA (33.9% of all adults) [2]—have prediabetes diabetes. Given the magnitude of these numbers, identifying those at risk and preventing or delaying onset of type 2 diabetes are critical to ending the pandemic.

Type 2 diabetes can be prevented or delayed through mitigation of modifiable risk factors, such as healthier eating, weight loss, and increased physical activity. Studies show lifestyle intervention as a sole modality, lifestyle intervention in combination with therapeutics, and whole-population approaches are all promising for type 2 diabetes prevention. Public health interventions using glycemic risk stratification to target individuals at “very high” risk (having obesity and impaired glucose tolerance (IGT)) and “high” risk (being overweight with IGT) have proven to significantly reduce conversion to type 2 diabetes [4]. Individuals in these categories have a fasting plasma glucose (FPG) > 100 mg/dL, HbA1c levels > 5.7%, and a 10-year diabetes incidence of 20–30% or more [5]. Individuals in lower risk tiers may be more appropriately targeted via risk counseling and whole-population strategies [5–7]. Population-level policies, systems, and environmental approaches, along with lifestyle intervention for those at high risk, are likely optimal to achieve the greatest level of impact [8].

The purpose of this review is to highlight the foundational and current research and translation studies which underlie high-risk population and whole-population strategies for type 2 diabetes prevention worldwide, with particular focus on the U.S. National Diabetes Prevention Program, as catalysts for global prevention efforts.

## Worldwide Evidence for Type 2 Diabetes Prevention Through Lifestyle Change

### Foundational Research

The scientific evidence for prevention through lifestyle changes is compelling based on a series of randomized control trials (RCTs), which found through intensive, structured, yearlong educational programs focused on moderate weight loss (5–7%), increasing self-efficacy around engagement in one’s health, and moderate increases in physical activity over time, it is possible to prevent or delay type 2 diabetes among those at very high and high risk. RCTs have been conducted in various settings among diverse racial and ethnic populations. Common elements include utilizing a group-based intervention and evaluating effectiveness in terms of increased physical activity and healthy eating, and improved clinical metrics, such as weight, body mass index (BMI), waist circumference, HbA1c, and blood glucose.

The Chinese Da Qing group-based RCT was the first and longest running of such studies. It began in 1986 [9] with a follow-up study conducted in 1997–2006. The study demonstrated a 43% incidence reduction in the intervention group after 14 years when compared to the control group. Type 2 diabetes was delayed by an average of 3.6 years [10], and the incidence of severe retinopathy and cardiovascular-related disease and events were reduced [10, 11]. After 30 years, 577 adults with IGT were followed from the original trial; the intervention group had a median delay in type 2 diabetes incidence of 3.96 years, an average increase in life expectancy of 1.44 years, and fewer cardiovascular disease and all-cause deaths, reduced incidence of cardiovascular events, and lower incidence of microvascular complications compared to the control group [12].

The U.S. Diabetes Prevention Program (DPP) was a three-arm RCT, which began in 1996 [13••]. The study found that a lifestyle change intervention focused on a 5–7% weight loss and a moderate increase in physical activity over one year achieved a 58% relative risk reduction in type 2 diabetes, and that use of metformin achieved a 31% reduction, when compared to a placebo [13••]. The DPP used intensive one-on-one counseling with a minimum of 50% racially and ethnically diverse individuals across both male and female genders at high risk for type 2 diabetes (elevated plasma glucose of 95 to 125 mmol/L or fasting glucose of 7.8 to 11.0 mmol/L) [13••]. A follow-up study, the U.S. Diabetes Prevention Program Outcomes Study (DPPOS), reported a 34% reduction in type 2 diabetes incidence 10 years after the completion of the DPP trial for the lifestyle change intervention arm [14], and a 27% reduction after 15 years (18% for the metformin arm), compared to the placebo group [15]. Furthermore, the study found that lifestyle change was cost-effective compared to a placebo, and that cumulative quality-adjusted life years (QALYs) gained over a 3-year timeframe were greater for lifestyle (6.81) than either metformin (6.69) or a placebo (6.67) [16].

The Finnish Diabetes Prevention Study (Finnish DPS), which began in 2001 [17], tested lifestyle intervention in a community-based primary health care setting, and designed and implemented a high-risk screening assessment for type 2 diabetes that is used worldwide called the Finnish Type 2 Diabetes Risk Score [18]. The study demonstrated a risk reduction of 58%, as well as a legacy effect of 43% reduction in type 2 diabetes incidence three years after completion of the study. The study has also been successfully translated in Greece with similar results [19].

The Japanese DPP, conducted in 2005 in male participants with IGT > 140 mg/dL, found a cumulative 4-year incidence of diabetes of 9.3% in the control group, versus 3.0% in the intervention group [20]. The Indian DPP-1 trial, conducted in 2006, was a community-based RCT involving 531 subjects with IGT across three intervention arms (one was given advice on lifestyle modification (LSM), one was treated with metformin (MET), and another was given LSM plus MET) and one control arm. A relative risk reduction of 26–29% after 30 months was similar in all three intervention arms [21]. Additional 3-year results suggest that both metformin and lifestyle change were cost-effective in preventing type 2 diabetes [22].

### Translational Research

Translational research, which examines how to best tailor key research findings into policy, program, or practice [23], further demonstrates that lifestyle interventions are feasible and effective in real-world settings [24]. A systematic review and meta-analysis of 28 US-based DPP translation studies found a mean weight loss of over 4% across 3797 high-risk participants and demonstrated cost-effectiveness in terms of program, materials, and staff costs [24].

The European DE-PLAN study (“Diabetes in Europe – Prevention using Lifestyle, Physical Activity, and Nutritional Intervention”), implemented in 17 countries, was a community-based 10-month translation of the Finnish DPS targeting those at high risk for type 2 diabetes [19]. DE-PLAN began in 2008 and used the Finnish Diabetes Risk Score to determine eligibility [19]. In 2018, study participants in Poland (*n* = 175) with increased risk for type 2 diabetes received 10 months of lifestyle counseling sessions, physical activity, and self-efficacy sessions [25••]. Participants with a higher starting BMI and a history of increased glucose were more likely to achieve the goal weight loss of ≥ 5% of initial body weight compared to those with lower risk [25]. A UK study had similar findings, also determining adults with obesity and higher HbA1c levels (6–6.4%) not only met the weight loss outcomes of the US DPP trial but also gained more QALYs than those with lower risk; the intervention was also determined to be cost-saving [26].

The Australian Good Ageing in Lahti Region Lifestyle (GOAL) Implementation program, based on the Finnish DPS, found positive associations between changes in self-efficacy and dietary behaviors and improvement in waist circumference, cholesterol levels, triglycerides, diastolic blood pressure, and FPG in the lifestyle intervention arm compared to the control group [27]. GOAL was further scaled up with over 10,000 participants via the Melbourne DPS with significant improvements in cardiovascular risk factors, waist circumference, BMI, and weight loss [28].

Alternative modalities of lifestyle change program delivery, such as virtual program delivery and telehealth, have the potential to reach millions of people, even in remote areas. Shortly after publication of the 2002 DPP research study, Tate et al. (2003) conducted the first RCT of a yearlong Internet-based diabetes prevention lifestyle change weight loss program alone vs. one with the addition of e-behavioral counseling [29]. The group receiving e-behavioral counseling submitted calorie and exercise information and received weekly e-mail behavioral counseling and feedback from a counselor for 12 months and lost 4.8% of original body weight compared to 2.2% among those receiving the Internet program only [29]. A 2013 text messaging study in India showed that mobile technology can have an impact on clinical outcomes, with cumulative incidence of type 2 diabetes at 2 years of 18% in the text messaging counseling group vs. 27% in the control group [30], and a sustained reduction in incidence after 5 years [31]. Similar studies have been conducted (Supplemental Table 1) to determine the effectiveness of various forms of virtual delivery, including delivery via television, social networking sites, and online. These published studies on virtual delivery found ~ 4% to 10% weight loss after virtual implementation of a yearlong diabetes prevention lifestyle change program. A summary of results is included in Supplemental Table 1.

The evidence is clear: lifestyle interventions can prevent or delay type 2 diabetes among various races, ethnicities, genders, and regions. Additionally, type 2 diabetes prevention program interventions continue to demonstrate cost-effectiveness/cost savings in real-world settings over time [32, 33].

## The US National DPP—a Case Study

Large-scale implementation of the US DPP trial’s lifestyle change program began in 2010, when Congress authorized the US Centers for Disease Control and Prevention (CDC) to establish and lead the National DPP in an effort to make the intervention broadly available to individuals at high risk [34]. The National DPP is a partnership of public and private organizations working to build a delivery system for the lifestyle intervention. It consists of four core elements: a trained workforce of lifestyle coaches; national quality standards supported by the CDC Diabetes Prevention Recognition Program (DPRP); a network of program delivery organizations sustained through coverage; and participant referral and engagement [35]. The National DPP lifestyle change program is based on evidence and key features of the US DPP trial that were shown to be successful: realistic weight loss goal after 12 months (minimum 5% of initial body weight), documentation of physical activity minutes (≥ 150 min per week), and attendance throughout the 12-month program, with an emphasis on self-efficacy that focuses on improving problem-solving skills, social supports, the use of built environments, and strategies to adapt to change [35]. The National DPP is the world’s largest translation of the US DPP study, having reached over 324,000 participants across > 3,000 organizations as of April 12, 2019 [36]. The DPRP is the quality assurance arm of the National DPP, developing evidence-based standards (DPRP Standards) and monitoring and evaluating participating organizations for fidelity and effectiveness of intervention delivery [37]. The DPRP Standards are updated every three years based on new dietary, physical activity, self-efficacy, delivery modality, and other type 2 diabetes prevention evidence. Through the DPRP, CDC awards either preliminary (based on participant attendance rather than outcomes) or full (meeting all DPRP Standards) recognition to successful organizations [37]. CDC recognition is widely accepted in the USA as an assurance of a quality type 2 diabetes prevention program and can result in insurance coverage for participants and reimbursement for program delivery organizations.

Public and private insurance coverage for type 2 diabetes prevention interventions is crucial to widespread adoption and participation in National DPP lifestyle change programs. Insurance coverage expands payment options, thereby reducing the burden of cost for participants. Currently, over 3.8 million public employees and dependents in 20 states have the National DPP lifestyle change program as a covered benefit and over 100 private employers and commercial plans include it as a covered benefit for their employees [38]. In 2018, the US Centers for Medicare & Medicaid Services (CMS) began coverage for eligible Medicare beneficiaries who participate in CDC-recognized programs and meet performance goals of the National DPP [40]. CMS provides reimbursement for participants meeting program goals such as 5% weight loss in organizations that have achieved either preliminary or full CDC recognition [39]. Eight states in the USA also provide Medicaid coverage for eligible beneficiaries [38].

Based on published translational research, feedback from stakeholder organizations, and gray literature scans (stakeholder materials and policies not found in peer-reviewed journals), CDC concluded that there was sufficient evidence to allow organizations delivering the National DPP lifestyle change program virtually to apply for CDC recognition. Thus, in order to expand availability and increase program participation (especially for those in rural or remote locations), the DPRP Standards were amended in January 2015 to allow online modes of delivery in addition to in-person. The Standards were amended again in February 2018 to also include telehealth and combination (in-person/virtual) delivery and new participant-level variables that include education level, enrollment source, and payer source. All virtual providers are held accountable to the same quality standards as in-person delivery organizations, including the provision of coaching services [37]. As of April 2019, CDC had 121 recognized virtual providers delivering the yearlong lifestyle change program to over 193,000 people [36].

### Key Findings from In-Person National DPP Delivery in the USA

DPRP data from the first 4 years (February 2012–January 2016) of the National DPP, describing the experience of 14,747 participants who attended 4 or more sessions of the lifestyle change program in 220 organizations, showed an average weight loss of 4.2%, with 35.5% of participants achieving ≥ 5% weight loss [40]. Participants reported an average 152 min per week of physical activity, with 41.8% meeting the physical activity goal of 150 min per week. Participants with longer retention and greater participation in the program and who were more physically active were more likely to have higher weight loss [40].

### Implementation of the National DPP: New Insights

#### Analysis Sample

**Fig. 1:**
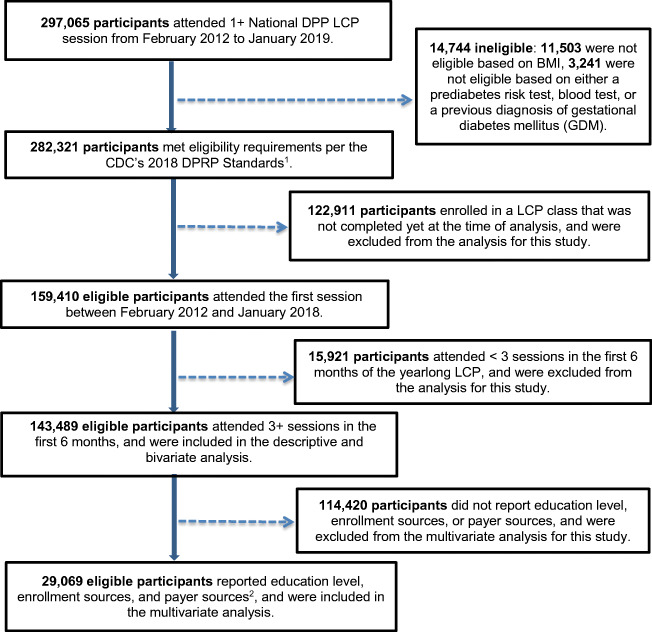
Flow chart for analysis sample. CDC = Centers for Disease Control and Prevention; DPRP = Diabetes Prevention Recognition Program; LCP = CDC-recognized lifestyle change program; National DPP = National Diabetes Prevention Program. ^1^Participant eligibility was based on BMI (≥ 25 kg/m^2^, or ≥ 23 kg/m^2^ if Asian American), a blood test indicating prediabetes, a CDC Prediabetes Screening Test or American Diabetes Association Type 2 Diabetes Risk Test, or a previous diagnosis of gestational diabetes mellitus. ^2^Education level, enrollment sources, and payer sources were not collected before February 2018

To further assess National DPP implementation success, we examined recent DPRP data and analyzed new variables (education level, enrollment source, and payer source) to assess their relationship with participant outcomes. More than 297,000 participants attended one or more lifestyle change program sessions between February 2012 and January 2019. For the purpose of this new analysis, 143,489 eligible participants across all program modalities (in-person, online, distance learning, and combination) who completed the yearlong lifestyle change program and attended ≥ 3 sessions in the first 6 months (analyzed participants) were included in descriptive analyses. Of those, 29,069 eligible participants who started the program in 2018 and reported new variables (education level, enrollment source, and payer source) were included in the multivariable analysis (Fig. 1).

#### Measures and Methods

Percent body weight change was the primary outcome of this analysis, calculated among participants with at least 2 documented body weights using the first (baseline) and last recorded weights. Average weight loss and physical activity minutes per week were calculated among participants who attended ≥ 3 sessions in the first 6 months and whose time from first session attended to last session attended was ≥ 9 months. CDC considers this threshold to be the minimum dose to begin seeing lifestyle and weight change that can impact type 2 diabetes [40].

Mantel-Haenszel chi-square tests of difference were used to assess bivariate associations between participants’ characteristics and duration of participation. Results were stratified by duration of participation (≥ 9 vs. < 9 months). Multiple logistic regression models were used to estimate the association between participants’ attendance and duration of participation and the likelihood of meeting the minimum 5% weight loss goal conditional on other factors. Adjusted odds ratios (AORs) in relation to a reference category were reported with their respective 95% confidence intervals (CIs). Results with *p* < 0.05 were considered statistically significant. All analyses were conducted using SAS, version 9.4.

## Results

Overall, about 60% of analyzed participants attended the lifestyle change via an online-only modality, 40% via an in-person only modality, and <  1% via distance learning only or combination modality (Table 1). About 40% of analyzed participants attended at least 17 lifestyle change sessions; 31% met the minimum 5% weight loss goal, and 45% met the average 150+ min per week of physical activity goal. Three quarters were females; over half were aged 45–64 years; over 60% were non-Hispanic whites; about half reported having 4 years of college or more; and over 70% had obesity (body mass index (BMI) ≥ 30 kg/m^2^).

**Table 1:** Characteristics and outcomes of National DPP participants who completed the yearlong lifestyle change program and attended ≥ 3 sessions in the first 6 months

Organization and participant characteristics	Total analyzed participants *n* = 143,489 (%)	Participants with < 9 months *n* = 99,046 (69%) (%)	Participants with ≥ 9 months *n* = 44,443 (31%) (%)	*p* value*
Organizations’ characteristics				< 0.001
Delivery modality				
Online only	85,362 (59.5)	68,137 (68.8)	17,225 (38.8)	
Distance learning only	471 (0.3)	234 (0.2)	237 (0.5)	
Combination	1,052 (0.7)	519 (0.5)	533 (1.2)	
In-person only	56,604 (39.5)	30,156 (30.5)	26,448 (59.5)	
Participants’ average weight loss (WL)				*< 0.001*
Percent WL at 12 months				
5%+	45,068 (31.4)	22,556 (22.8)	22,512 (50.7)	
< 5%	98,421 (68.6)	76,490 (77.2)	21,931 (49.3)	
Participants’ attendance and physical activity (PA) goal				
Number of sessions attended				*< 0.001*
17+ sessions	56,983 (39.7)	17,741 (17.9)	39,242 (88.3)	
< 17 sessions	86,506 (60.3)	81,305 (82.1)	5,201 (11.7)	
Meeting PA goal (average 150+ min/week)				*< 0.001*
Met	60,348 (45.0)	36,503 (40.2)	23,845 (54.9)	
Not met	73,837 (55.0)	54,260 (59.8)	19,577 (45.1)	
Participants’ characteristics				
Age (years)				*< 0.001*
18–44	43,914 (30.6)	36,577 (36.9)	7,337 (16.5)	
45–64	78,178 (54.5)	51,751 (52.3)	26,427 (59.5)	
65+	21,397 (14.9)	10,718 (10.8)	10,679 (24.0)	
Sex				*< 0.001*
Male	35,401 (24.7)	25,564 (25.8)	9,837 (22.1)	
Female	107,990 (75.3)	73,437 (74.1)	34,553 (77.8)	
Race/ethnicity				0.09
Hispanic	12,898 (9.0)	9,138 (9.2)	3,760 (8.5)	
Non-Hispanic Black	17,088 (11.9)	11,477 (11.6)	5,611 (12.6)	
Non-Hispanic other^a^	20,846 (14.5)	14,790 (14.9)	6,056 (13.6)	
Non-Hispanic White	92,657 (64.6)	63,641 (64.3)	29,016 (65.3)	
Education level				0.06
High school or less	3,410 (10.1)	2,102 (9.5)	1,308 (11.2)	
Some college or technical school	12,537 (37.1)	8,598 (38.8)	3,939 (33.8)	
College 4 years or more	17,878 (52.9)	11,480 (51.8)	6,398 (54.9)	
Payer type				*< 0.001*
Grant funding	3,302 (7.8)	1,363 (5.2)	1,939 (11.9)	
Public insurer (Medicare or Medicaid)	208 (0.5)	83 (0.3)	125 (0.8)	
Employer or private insurer	36,985 (86.8)	24,137 (91.7)	12,848 (78.9)	
Self-pay or other	2,114 (5.0)	748 (2.8)	1,366 (8.4)	
Enrollment Source				*< 0.001*
Health care providers^b^	2,035 (5.2)	780 (3.1)	1,255 (8.9)	
Community-based organizations or community health workers	515 (1.3)	130 (0.5)	385 (2.7)	
Self or family/friends	2,431 (6.2)	1,382 (5.6)	1,049 (7.4)	
Employers or insurers	29,272 (75.0)	20,045 (80.5)	9,227 (65.4)	
Media or other	4,757 (12.2)	2,570 (10.3)	2,187 (15.5)	
Body mass index (BMI) at enrollment				*< 0.001*
Overweight^c^	39,226 (27.3)	26,373 (26.6)	12,853 (28.9)	
Obesity^d^	104,263 (72.7)	72,673 (73.4)	31,590 (71.1)	

*p value was based on the Mantel-Haenszel chi-square test of the difference between participants’ characteristics and duration of participation. Italicized values indicate statistical significance at *p* < 0.05

^a^Non-Hispanic other race/ethnicity includes non-Hispanic Asian, non-Hispanic Native Hawaiian or other Pacific Islander, non-Hispanic American Indian or Alaska Native, or multiracial, or not reported

^b^Includes primary care provider/office or specialist (e.g., MD, DO, PA, NP, or other staff at the provider’s office), or non-primary care health professional (e.g., pharmacist, dietitian)

^c^BMI, 23–29 for Asian or 25–29 for other race/ethnicity

^d^BMI, ≥ 30

Weight loss success was significantly higher among those who attended ≥ 17 sessions (AOR 3.2), those who stayed in the program for ≥ 9 months (AOR 1.3), and those with ≥ 150 min of physical activity per week (AOR 1.7) (Table 2). Participants aged 45–64 and ≥ 65 years had 3.2 and 1.6 times, respectively, the odds of meeting the 5% weight loss goal than those aged 18–44. Females, non-Hispanic blacks, and those with obesity were slightly less likely to meet the 5% weight loss goal than males, non-Hispanic whites, and those overweight (BMI between 25 and 29.9 kg/m^2^).

**Table 2:** Adjusted odds ratios for meeting the 5%+ weight loss goal among analyzed participants, and by participation duration

Predictors	Total analyzed participants (*n* = 29,069)	Participants with < 9 months (*n* = 19,788)	Participants with 9+ months (*n* = 9281)
	AOR (95% CI)	AOR (95% CI)	AOR (95% CI)
Organizations’ characteristic			
Delivery modality			
Online only	0.9 (0.8–1.2)	1.1 (0.7–1.6)	0.9 (0.7–1.2)
Distance learning only	–	–	–
Combination	1.6 (0.7–4.0)	–	2.6 (0.9–7.5)
In-person only (ref)	1.00	1.00	1.00
Participants’ attendance and physical activity (PA) goal			
Number of session attendance			
17+ sessions	3.2 (3.0–3.4)***	3.4 (3.1–3.6)***	2.6 (2.3–3.0)***
< 17 sessions (ref)	1.00	1.00	1.00
Duration of participation			
9+ months	1.3 (1.2–1.4)***	–	–
< 9 months (ref)	1.00	–	–
Meeting PA goal (average + 150 min/week)			
Met	1.7 (1.6–1.8)***	1.7 (1.6–1.9)***	1.7 (1.5–1.8)***
Not met (ref)	1.00	1.00	1.00
Participants’ characteristics			
Age (years)			
18–44 (ref)	1.00	1.00	1.00
45–64	3.2 (3.0–3.4)**	1.2 (1.0–1.3)	1.0 (0.9–1.1)
65+	1.6 (1.5–1.8)***	1.7 (1.5–2.0)***	1.4 (1.2–1.6)***
Sex			
Male (ref)	1.00	1.00	1.00
Female	0.8 (0.7–0.8)***	0.7 (0.6–0.7)***	0.9 (0.8–1.0)
Race/ethnicity			
Hispanic	0.9 (0.8–1.0)	0.9 (0.8–1.0)	0.8 (0.7–0.9)*
Non-Hispanic Black	0.8 (0.7–0.9)*	0.9 (0.7–1.0)	0.8 (0.7–0.9)*
Non-Hispanic other^a^	0.9 (0.8–1.0)	0.9 (0.8–1.0)	0.9 (0.8–1.1)
Non-Hispanic White (ref)	1.00	1.00	1.00
Education level			
High school or less (ref)	1.00	1.00	1.00
Some college or technical school	0.9 (0.8–1.0)	0.9 (0.8–1.0)	1.0 (0.8–1.1)
College 4 years or more	0.9 (0.8–1.0)	0.8 (0.7–0.9)***	1.0 (0.9–1.1)
Payer type			
Grant funding (ref)	1.00	1.00	1.00
Public insurer (Medicare or Medicaid)	1.1 (0.7–1.7)	1.4 (0.5–3.9)	0.9 (0.5–1.5)
Employer or private insurer	1.2 (1.0–1.5)	1.5 (1.0–2.0)	1.0 (0.7–1.3)
Self-pay or other	1.4 (1.0–1.8)	2.3 (1.5–3.7)*	1.0 (0.7–1.4)
Enrollment source			
Health care providers^b^ (ref)	1.00	1.00	1.00
Community-based organizations or community health workers	0.7 (0.4–1.1)	0.1 (0.1–1.1)	0.8 (0.5–1.3)
Self or family/friends	0.9 (0.7–1.2)	1.0 (0.7–1.6)	0.8 (0.6–1.1)
Employers or insurers	0.8 (0.6–1.0)	0.9 (0.6–1.3)	0.8 (0.6–1.0)
Media or other	0.9 (0.7–1.1)	0.9 (0.6–1.4)	0.8 (0.6–1.1)
Body mass index (BMI) at enrollment			
Overweight^c^	1.1 (1.1–1.2)**	1.1 (0.9–1.1)	1.2 (1.1–1.3)***
Obesity^d^ (ref)	1.00	1.00	1.00

Italicized values indicate statistical significance (*p < 0.05, **p < 0.01, ***p < 0.001). Data are presented as AOR (95% CI) based on multiple logistic regression. Analyzed participants include eligible participants who completed the yearlong lifestyle change program and attended ≥ 3 sessions in the first 6 months

^a^Non-Hispanic other race/ethnicity includes non-Hispanic Asian, non-Hispanic Native Hawaiian or other Pacific Islander, non-Hispanic American Indian or Alaska Native, multiracial, or not reported

^b^Includes primary care provider/office or specialist (e.g., MD, DO, PA, NP, or other staff at the provider’s office), or non-primary care health professional (e.g., pharmacist, dietitian)

^c^BMI, 23–29 for Asian or 25–29 for other race/ethnicity

^d^BMI, ≥ 30

This analysis is subjected to some limitations. First, biometric data (weight and physical activity minutes) was self-reported either by CDC-recognized organizations or participants themselves. However, lifestyle coaches were provided guidance to use the same scale at each session for recording participants’ body weight to ensure consistency. Second, calculation of percent weight loss was based on first and last recorded weight. Participants who lost more weight might be more likely to stay in the program longer and continue to lose more weight by the end of the program than those who dropped out early. Third, CDC’s DPRP began requiring organizations to submit information on participants’ education level, enrollment source, and payer source in February 2018; thus, our analyses were limited to a small sample of participants who started the program in 2018 (~ 10% of the total National DPP participants), as the remaining participants had not yet had the opportunity to participate in the program for a year prior to our study. Lastly, the analysis only included eligible participants based on CDC’s DPRP Standards, so it may not be generalizable to ineligible participants who may also benefit from this program.

### Participation in Lifestyle Change Intervention

Successful expansion of lifestyle change interventions relies on sufficient enrollment and retention. A recent analysis of 2016–2017 U.S. National Health Interview Survey data showed that 73.5% of adults with overweight or obesity with diagnosed prediabetes and 50.6% of adults with overweight or obesity and elevated American Diabetes Association (ADA) risk scores reported receiving risk reduction advice or referrals to risk reduction activities from their health care providers [41•]. Of those referred, only one third reported engagement in risk-reducing activities in the past year, and less than 3% reported participating in a type 2 diabetes prevention program. The key drivers for engagement in risk-reducing activities and programs included receiving advice from a health professional, having higher education, having insurance, being non-white race/ethnicity, and being middle aged. This study underscores the need for research from fields such as behavioral economics, human-centered design, and habit formation, to understand how to best engage people at high risk in type 2 diabetes prevention programs. Other approaches for systems change include the establishment of referral processes from clinical health care providers to community-based implementation programs, and media and marketing strategies to drive traffic to such programs. A recent study found that primary care providers in the USA who were aware of the National DPP lifestyle change program and the Prevent Diabetes STAT: Screen, Test, and Act Today™ Toolkit developed by CDC and the American Medical Association (AMA) were more likely to screen patients for prediabetes and make referrals to CDC-recognized organizations offering the National DPP. Those who used electronic health records were also more likely to screen, test, and refer [42].

Offering diabetes prevention programs via businesses and worksites could help lower employee health care costs and increase QALYs, and is an important systems approach still in need of broader consideration among employers [43]. Evidence-based programs within worksite wellness programs and health-based interventions within worksites are increasing in scope in the USA, and seem to be slowly gaining traction elsewhere in the world as well. To assist employers and insurers in determining the feasibility of providing the National DPP lifestyle change program or including it as part of their employees’ insurance benefits, CDC developed the Diabetes Prevention Impact Toolkit [43]. The toolkit helps estimate the cost per employee and the associated cost savings related to offering the National DPP lifestyle change program, including estimating the employee’s QALYs gained. A summary of recent peer-reviewed literature on employer-based diabetes prevention programs in the USA found that greater weight loss and maintenance of weight loss were achieved among worksites that implemented the National DPP lifestyle change program compared to worksites that implemented other interventions [44].

Another key approach to increasing participation focuses on raising awareness of both prediabetes as a serious condition and of type 2 diabetes prevention activities. This is a challenge, as many providers are not screening or testing patients for prediabetes when considering its risk factors (obesity, overweight, glycemic range, and cardiovascular risks) In the USA, CDC, ADA, and AMA partnered with the Ad Council to launch the nation’s first national public service campaign about prediabetes [45] which encourages people to take a short online test at DoIHavePrediabetes.org to learn their prediabetes risk. From the campaign’s launch on January 21, 2016, through March 31, 2019, 2.5 million people have completed the online risk test [46].

### Whole-Population Approaches

Because of the widespread prevalence of type 2 diabetes and its risk factors, lifestyle change programs for the highest-risk people alone cannot sufficiently impact the diabetes pandemic without approaches that support prevention efforts across whole systems and communities. Larger-scale, population-wide prevention strategies, such as environmental, policy, cost reimbursement, and health marketing/awareness efforts, are therefore needed. Several whole-population approaches have been implemented in the USA. Many focus on socioeconomic policy involving nutrition regulation such as menu labeling; subsidies to increase the affordability of fruits and vegetables, particularly in rural or hard-to-reach areas [47, 48]; sugar-sweetened beverage tax and decreased added sugars; and increased whole grains, fibers, nuts, and legumes and elimination of trans fats (or trans fatty acids) as recommended by the American Heart Association [49]. Globally, supported by WHO, clear nutrition “front-of-pack labeling” has been found to improve dietary habits and reduce cardiovascular complications [50].

A systematic review found strong evidence in Europe for implementing multiple policies simultaneously, including taxes on unhealthy food, subsidies for healthy food, trans fat elimination, and trade agreements with supportive countries who implement similar taxation and food policies [51••]. A 2014 systematic review of the evidence behind food taxation and food subsidies (government/local investments in healthy food) found that both should be implemented in tandem at a minimum rate of 10 to 15% to ensure the unhealthy foods are less accessible and healthy foods/beverages are more accessible to purchasers [52]. Similarly, Colchero et al. studied the effect of taxing sugar-sweetened beverages in stores in Mexico and found reductions in purchases of the taxed beverages associated with increases in purchases of untaxed beverages [53].

Environmental changes such as targeting the built environment and community planning efforts also show promise in reaching large populations. These approaches include the expansion or building of walking, biking, and hiking trials, and other “safe” routes [54]. A systematic review evaluating the effect of built environment policies on obesity-related outcomes across the USA, Canada, Chile, the UK, and New Zealand found that physical activity-related policies had a stronger impact when they involved improvements to transportation infrastructure. These improvements included creating more structural access such as building cycling lanes and park trails [55].

A possible limitation of whole-population approaches is that researchers have to rely largely on modeling studies or intermediate outcomes to determine impact. Short-term and longitudinal impact testing involving actual health and economic trends is more difficult, but critical to assessing intervention success. Also critical is understanding the contexts in which whole-population approaches are most effective.

## Conclusion

To achieve large-scale type 2 diabetes prevention, interventions directed to both high-risk populations and the general population are necessary. There is strong evidence for the prevention of type 2 diabetes from RCTs and subsequent translation studies in which people at high risk engage in a structured lifestyle intervention that addresses nutrition, physical activity, and behavior change strategies resulting in a weight loss of ≥ 5%. Whole-population strategies also show promise in reaching large numbers of people and include multi-sector and multi-policy approaches, most successfully in combination. These approaches include taxation of unhealthy foods, enhancing the built environment, addressing food accessibility, offering worksite wellness, and raising awareness of prediabetes among health care providers and the general public.

Large-scale implementation of what has been proven to work, including alternate delivery approaches for type 2 diabetes prevention programs to reach disparate and geographically isolated populations, is needed. Continued examination of outcomes and program effectiveness is needed to refine global prevention efforts. Fortunately, there is much evidence worldwide that type 2 diabetes prevention or delay is attainable, and that prevention strategies can be adapted across cultures and environments. Success will require a combination of policy, systems, environmental, and health marketing/awareness approaches with effective interventions for high-risk populations and partnerships across sectors. Based on the data showing the impact of diabetes it warrants being prioritized to protect the public’s health around the world and curb the increasing burden of diabetes.

## Electronic supplementary material

ESM 1 (DOCX 49 kb)
