# Supplementary material for: Public Health Approaches to Type 2 Diabetes Prevention: the US National Diabetes Prevention Program and Beyond
Source: Curr Diab Rep. 2019 Aug 5;19(9):78. doi: 10.1007/s11892-019-1200-z (PMC6682852; doi:10.1007/s11892-019-1200-z)
Supplement: Supplementary file 1 — (DOCX 49 kb) [file 11892_2019_1200_MOESM1_ESM.docx]

**Supplemental Table 1.** Studies of Virtual Type 2 Diabetes Prevention Program Delivery

| **Citation** | **Evidence for Study** | **Results** | **Weight**  **Loss or Type 2 Diabetes Incidence Reduction** |
| --- | --- | --- | --- |
| Tate DF, Jackvony EH, Wing RR. Effects of internet behavioral counseling on weight loss in adults at risk for type 2 diabetes: a randomized trial. [JAMA](http://jama.jamanetwork.com/article.aspx?articleid=196319). 2003;289(14):1833-1836. | Based on the 2002 DPP clinical trial | RCT of 92 overweight adults (mean BMI 33.1) at high risk for type 2 diabetes showed that the group receiving e-behavioral counseling in addition to the internet weight loss program lost significantly more weight than the internet-only program. | 4.8% weight loss in e-counseling + internet group vs. 2.2% in internet program-only group |
| Ackermann, R. T., Sandy, L. G., Beauregard, T., Coblitz, M., Norton, K. L. and Vojta, D. (2014), A randomized comparative effectiveness trial of using cable television to deliver diabetes prevention programming. [Obesity.](http://onlinelibrary.wiley.com/doi/10.1002/oby.20762/full) 22(7):1601-1607. | Benchmarked against CDC DPRP Standards | A study of 306 individuals in two US cities, sponsored by UnitedHealth Group and Comcast Cable. Studied in-home delivery of a diabetes prevention program in a reality television format, offered with or without online behavioral support tools, can achieve weight loss consistent with past implementation studies of in-person programs using similar content. | 4.9% mean weight loss amongst participants completing 9 or more sessions |
| Sepah CS, Jiang L, Peters AL. (2104). Translating the diabetes prevention program into an online social network: Validation against CDC standards. [The Diabetes Educator](http://tde.sagepub.com/content/40/4/435.full). 40(4):435-443. | Benchmarked against CDC DPRP Standards | Omada Health study to evaluate the efficacy of *Prevent*, an online social network-based translation of the DPP lifestyle intervention, against CDC DPRP Standards’ outcomes and outcomes of other diabetes prevention program translations. | 5.2% average weight loss at 12 months |
| [Ma J](http://www.ncbi.nlm.nih.gov/pubmed?term=Ma%20J%5BAuthor%5D&cauthor=true&cauthor_uid=23229846), [Yank V](http://www.ncbi.nlm.nih.gov/pubmed?term=Yank%20V%5BAuthor%5D&cauthor=true&cauthor_uid=23229846), [Xiao L](http://www.ncbi.nlm.nih.gov/pubmed?term=Xiao%20L%5BAuthor%5D&cauthor=true&cauthor_uid=23229846), [Lavori PW](http://www.ncbi.nlm.nih.gov/pubmed?term=Lavori%20PW%5BAuthor%5D&cauthor=true&cauthor_uid=23229846), [Wilson SR](http://www.ncbi.nlm.nih.gov/pubmed?term=Wilson%20SR%5BAuthor%5D&cauthor=true&cauthor_uid=23229846), [Rosas LG](http://www.ncbi.nlm.nih.gov/pubmed?term=Rosas%20LG%5BAuthor%5D&cauthor=true&cauthor_uid=23229846), [Stafford RS](http://www.ncbi.nlm.nih.gov/pubmed?term=Stafford%20RS%5BAuthor%5D&cauthor=true&cauthor_uid=23229846). Translating the Diabetes Prevention Program lifestyle intervention for weight loss into primary care: a randomized trial**.** [JAMA Intern Med.](http://www.ncbi.nlm.nih.gov/pubmed/23229846) 2013 Jan 28;173(2):113-21. | Based on the DPP clinical trial and Finnish DPS | A primary care-based RCT designed to evaluate the effectiveness of 2 adapted diabetes prevention lifestyle interventions among overweight or obese adults with prediabetes: a coach-led, face-to-face group intervention and a self-directed DVD intervention. | 7% weight loss goal at 15 months in 37.0% of participants in coach-led intervention, 35.9% (P = .004) in the self-directed intervention, vs. 14.4% in usual care group |
| Marrero D, Palmer K, Rost S, Frederick A, Miller-Kovach K, Saha C. Using Weight Watchers approach to lifestyle modification to reduce risk for type 2 diabetes. International Diabetes Federation. Melbourne, Australia 2013. | Modified Weight Watchers program with curriculum approved by CDC and benchmarked against CDC DPRP Standards | Weight Watchers conducted a clinical trial in 226 adults with prediabetes randomized to an experimental intervention using the Weight Watchers program with an introductory session added for persons with prediabetes or a control intervention (consists of educational materials and brief counseling). Intervention subjects were “mainstreamed” into Weight Watchers programs in their communities and/or online. Thirty percent of participants chose online only. | 6.2% average weight loss in intervention group vs. 1.0% in control group |
| Ross K, Kaufman ND. Automating coaching: Technology delivers proven weight loss intervention at affordable cost while minimizing personnel time. Presented at the Care Continuum Alliance conference, Oct. 2013. Provided by DPS Health. | Based on the DPP clinical trial | Evaluation of a yearlong online version of a diabetes prevention program called Virtual Lifestyle Management (VLM) in 385 Government Employees Health Association (GEHA) insurance plan members with a BMI > 27.5 and obesity-related comorbidity (e.g. abnormal glycemia, sleep apnea) or obese (BMI > 30) with or without comorbidity. | 3.7-10% |
| [Kanaya AM](http://www.ncbi.nlm.nih.gov/pubmed?term=Kanaya%20AM%5BAuthor%5D&cauthor=true&cauthor_uid=22698027), [Santoyo-Olsson J](http://www.ncbi.nlm.nih.gov/pubmed?term=Santoyo-Olsson%20J%5BAuthor%5D&cauthor=true&cauthor_uid=22698027), [Gregorich S](http://www.ncbi.nlm.nih.gov/pubmed?term=Gregorich%20S%5BAuthor%5D&cauthor=true&cauthor_uid=22698027), [Grossman M](http://www.ncbi.nlm.nih.gov/pubmed?term=Grossman%20M%5BAuthor%5D&cauthor=true&cauthor_uid=22698027), [Moore T](http://www.ncbi.nlm.nih.gov/pubmed?term=Moore%20T%5BAuthor%5D&cauthor=true&cauthor_uid=22698027), [Stewart AL](http://www.ncbi.nlm.nih.gov/pubmed?term=Stewart%20AL%5BAuthor%5D&cauthor=true&cauthor_uid=22698027). The Live Well, Be Well study: a community-based, translational lifestyle program to lower diabetes risk factors in ethnic minority and lower-socioeconomic status adults. [Am J Public Health.](http://www.ncbi.nlm.nih.gov/pubmed/22698027) 2012 Aug;102(8):1551-8. | Based on the DPP clinical trial and the Finnish DPS | RCT to evaluate a *Live Well, Be Well*, a lifestyle program designed for lower-socioeconomic status (SES) and ethnic minority adults, primarily through telephone-based counseling. | Intervention group lost 2 pounds on average more than control group |
| Ramachandran et al.,  Effectiveness of mobile phone messaging in prevention of type 2 diabetes by lifestyle modification in men in India: a prospective, parallel-group, randomised controlled trial. Lancet Diabetes Endocrinol. 2013 Nov;1(3):191-8. | Based on the DPP clinical trial | Included 537 participants with IGT that were randomly assigned to a mobile phone messaging intervention group or to standard care group. At baseline, all men received personalized education and motivation regarding healthy lifestyle and written information about diet and physical activity. The text messaging group received additional educational and positive lifestyle texts twice a day. | Cumulative incidence of type 2 diabetes at two years was 18% in the text group, versus 27% in the control group |
